# Epidemiology of invasive pneumococcal infections: manifestations, incidence and case fatality rate correlated to age, gender and risk factors

**DOI:** 10.1186/s12879-016-1648-2

**Published:** 2016-08-03

**Authors:** Erik Backhaus, Stefan Berg, Rune Andersson, Gunilla Ockborn, Petter Malmström, Mats Dahl, Salmir Nasic, Birger Trollfors

**Affiliations:** 1 Department of Infectious Diseases, Skaraborg Hospital, 54185 Skövde, Sweden; 2 Queen Silvia Children’s Hospital, Sahlgrenska University Hospital, Gothenburg, Sweden; 3 Department of Infectious Diseases, Institute of Biomedicine, Sahlgrenska Academy, Gothenburg University, Gothenburg, Sweden; 4 Department of Infectious Diseases, Södra Älvsborg Hospital, Borås, Sweden; 5 Department of Infectious Diseases, Norra Älvsborg Hospital, Trollhättan, Sweden; 6 Department of Emergency Medicine, Kungälv Hospital, Kungälv, Sweden; 7 Research and Development Center, Skaraborg Hospital, Skövde, Sweden

**Keywords:** *Streptococcus pneumoniae*, Pneumococcal disease, Meningitis, Incidence, Manifestations, Predisposing factors, Mortality

## Abstract

**Background:**

Incidence, manifestations and case-fatality rate (CFR) of invasive pneumococcal disease (IPD) vary with age and comorbidities. New vaccines, changing age distribution, prolonged survival among immunocompromised patients and improved sepsis management have created a need for an update of basic facts to inform vaccine recommendations.

**Methods:**

Age, gender and comorbidities were related to manifestations and death for 2977 consecutive patients with IPD in a Swedish region with 1.5 million inhabitants during 13 years before introduction of pneumococcal conjugate vaccines (PCV) in the infant vaccination program. These data were related to population statistics and prevalence of several comorbidities, and compared with two previous studies giving a total follow-up of 45 years in the same area.

**Results:**

The annual incidence was 15/100,000 for any IPD and 1.1/100,000 for meningitis; highest among elderly followed by children < 2 years. It was 2238/100,000 among myeloma patients, followed by chronic lymphatic leukemia, hemodialysis and lung cancer, but not elevated among asthma patients. CFR was 10 % among all patients, varying from 3 % below 18 years to 22 % ≥ 80 years. During 45 years, the IPD incidence increased threefold and CFR dropped from 20 to 10 %. Meningitis incidence remained stable (1.1/100,000/year) but CFR dropped from 33 to 13 %. IPD-specific mortality decreased among children <2 years from 3.1 to 0.46/100,000/year but tripled among those ≥65 years.

**Conclusions:**

IPD incidence and CFR vary widely between age and risk groups and over time even without general infant vaccination. Knowledge about specific epidemiological characteristics is important for informing and evaluating vaccination policies.

## Background

*Streptococcus pneumoniae* causes a broad spectrum of diseases from otitis media and sinusitis, to nonbacteremic pneumonia and, finally, to invasive pneumococcal diseases (IPD; (bacteremic pneumonia, septicemia with unknown focus and meningitis).

Individuals at extremes of age, and with various comorbidities run a higher risk of IPD [1–5]. Socioeconomic factors, tobacco and alcohol abuse also influence the incidence. Increasing numbers of patients living longer with multiple chronic diseases may change both IPD incidence and outcome. Improved management of sepsis may increase survival [6, 7]. Differences in incidence and case fatality rate (CFR) between countries at comparable socioeconomic levels [7], and between different time periods in the same country [8], might reflect an unequal distribution, both over time and geographically, of clones and serotypes with different virulence. However, such differences should be interpreted with caution [9], because of selection bias due to the impact of different blood culturing practices. When fewer blood cultures are performed the incidence seems lower, and if only severe cases are blood cultured, CFRs appear higher.

Although a polysaccharide vaccine (PPV23) had been available for risk groups > 2 years for almost 40 years [10, 11], it was not until widespread vaccination of infants with pneumococcal conjugate vaccines (PCV’s), that IPD incidence went down [12, 13] both among vaccinated children and unvaccinated adults in many countries. Since then, PCV13 (Prevnar13®), has become recommended, for example in the US, among immunocompromized adults [14] and adults aged ≥65 years, administered in series with PPV23 [15]. Since serotype distribution varies geographically and over time [8], both with and without the selective pressure of vaccination, continued success cannot be taken for granted in a long term perspective [16].

In Sweden, at the time of the study (1996–2008) PPV23 was recommended for certain risk groups [17] but largely underused. PCV was used in certain high risk groups but had not yet been introduced in the general childhood vaccination program. Gothenburg, with a population of 600,000, is the second largest city in Sweden, and capital of the Västra Götaland region with 1.5 million inhabitants. Incidence, manifestations and risk factors of IPD have been studied in the Gothenburg area in two earlier studies [5, 18] during 32 years, using the same study protocol, performed by a part of the present research team (Birger Trollfors, Mats Dahl).

The primary objective of this study was to investigate incidence, risk factors, manifestations, CFR and disease severity for all patients with IPD in the Västra Götaland Region during 13 years before introduction of PCV in the general childhood vaccination program, in January 2009. Secondary objectives were to compare greater Gothenburg with smaller cities and rural areas in the region, explore long-term trends over 45 years, and to estimate the relative risk for morbidity and mortality from different IPD manifestations in different age groups and for patients with specific comorbidities, in order to identify those with the highest potential benefit from vaccination.

## Methods

IPD was defined as an infectious episode during which pneumococci were isolated from normally sterile body fluids. All episodes of IPD during 1996–2008 in the Västra Götaland region (mean population 1,512,233) in Sweden were identified by the five microbiological laboratories serving all hospitals in the area. Data concerning age, sex, home municipality, manifestations, duration of hospitalization, death and sequelae were retrieved retrospectively from the medical records. Demographic information concerning population by municipality, age and sex in the region for each year retrieved from Statistics Sweden (SCB) were used to calculate incidence. Data on comorbidities, defined as the presence of co-existing medical conditions known or suspected to be related to an increased risk to get IPD, were also retrieved from records (as displayed in Table 1). CD4 counts at time of IPD were known for all patients with known HIV infection. Data on smoking was excluded since this information was missing in >50 %.

**Table 1:** Predisposing factors in 2977 patients with invasive pneumococcal disease: proportion, incidence rates and risk of death

Predisposing Factor	No. of episodes (%)	Died (No.)	CFR (%)	RR of death (95 % CI)	No. of Pat. with Factor^c^	Incidence^d^ (No./100,000/y.)	RR to get IPD (95 % CI)
Cardiovascular disease	720 (24)	126	18	2.35 (1.90–2.92)^a^			
Pulmonary disease	531 (18)	51	10	0.97 (0.73–1.29)			
- COPD	307 (10)	38	12	1.29 (0.94–1.78)	49,000	48	3.52 (3.12–3.98)^a^
- Asthma	145 (5)	4	3	0.27 (0.10–0.71)^b^	130,000	9	0.57 (0.48–0.68)^b^
Malignancy	485 (16)	87	18	2.16 (1.71–2.72)^a^	72,000	52	4.09 (3.69–4.52)^a^
- Haematological	257 (9)	35	14	1.43 (1.03–1.99)^a^	4900	403	29.16 (25.66–33.13)^a^
- - Myeloma	128 (4)	23	18	1.89 (1.28–2.78)^a^	440	2238	154.37 (132.51–179.84)^a^
- - Chronic Lymphatic Leukemia	53 (2)	4	8	0.76 (0.29–1.96)	950	429	28.86 (22.13–37.63)^a^
- Solid tumors	158 (5)	50	32	3.66 (2.82–4.73)^a^	67,200	18	1.26 (1.07–1.48)^a^
- - Lung	52 (2)	21	40	4.33 (3.05–6.13)^a^	1200	333	22.40 (17.11–29.33)^a^
- - Breast	23 (1)	0	0	n.a.	14,600	12	0.81 (0.53–1.22)
- - Colon	22 (1)	4	18	1.85 (0.76–4.53)	4600	37	2.44 (1.61–3.72)^a^
- - Prostate	46 (2)	5	11	1.10 (0.48–2.54)	11,900	30	1.99 (1.49–2.67)^a^
Diabetes mellitus	336 (11)	36	11	1.10 (0.79–1.52)	60,500	43	3.18 (2.83–3.57)^a^
Autoimmune Disease	227 (8)	26	11	1.17 (0.80–1.72)			
- Rheumatoid Arthritis	80 (3)	8	10	1.01 (0.52–1.97)	8500	72	4.91 (3.93–6.14)^a^
- Polymyalgia rheumatica	44 (1)	7	16	1.63 (0.82–3.23)			
- Systemic Lupus Erythematosus	23 (1)	2	9	0.88 (0.23–3.32)	830	213	14.19 (9.64–21.28)^a^
Liver disease	99 (3)	11	11	1.13 (0.64–1.99)			
Renal disease	109 (4)	17	16	1.61 (1.03–2.53)^a^			
- Haemodialysis	17 (1)	5	29	3.01 (1.43–6.34)^a^	385	340	22.56 (14.15–35.98)^a^
- Peritoneal dialysis	5 (0)	1	20	2.03 (0.35–11.75)	137	281	18.57 (7.85–43.96)^a^
Immune deficiency	93 (3)	7	8	0.76 (0.37–1.55)			
- HIV	13 (0)	1	8	0.78 (0.12–5.13)	407	246	16.30 (9.53–27.87)^a^
- Bone Marrow Transplant	20 (1)	2	10	1.01 (0.27–3.79)			
- Hypogammaglobulinemia	22 (1)	0	0				
- MGUS	26 (1)	2	8	0.59 (0.16–2.26)	17,000	12	0.81 (0.56–1.19)
Immunosuppressive treatment	279 (9)	36	13	1.35 (0.97–1.87)			
Asplenia	41 (1)	5	12	1.24 (0.54–2.84)	1500	210	14.08 (10.38–19.10)^a^
Alcohol Dependency	220 (7)	23	10	1.06 (0.71–1.59)			
≥ 1 predisposing factor^e^	1994 (67)	257	12.9	3.43 (2.45–4.81)^a^			
All Episodes	2977 (100)	294	9.9		1,512,233	15	

*n.a* not applicable

^a^Significantly higher relative risk (RR) to to die respectively to get IPD within 30 days from culture for a patient with this risk factor compared to all patients without this risk factor

^b^Significantly lower RR to die respectively to get IPD among asthma patients. The risk to die remainded significantly lower after correcting for age, sex and comorbidity. See text for details

^c^Estimated average no of patients living in the area based on estimated prevalence

^d^Estimated annual incidence among patients living with factor

^e^All patients with at least one risk factor. CFR among patients without any risk factor was 3.8 % (37/983)

Manifestations were defined as follows: Pneumonia or sinusitis required verification by X-ray or autopsy. Meningitis required isolation of pneumococci from cerebrospinal fluid (CSF) or positive blood cultures in combination with clinical symptoms and CSF cell counts. Septic arthritis required isolation of pneumococci from synovial fluid. Septicemia from an unknown focus was diagnosed when no focal infection could be identified. Osteomyelitis, otitis media, cellulitis and bronchitis were diagnosed clinically. Patients with mixed clinical diagnoses (of IPD) were handled as follows: Patients with meningitis and another manifestation were classified as meningitis, patients with pneumonia and some other non-meningitic manifestation were classified as pneumonia, and the remaining patients who neither had meningitis nor pneumonia were classified as described above. The remaining patients were classified as “sepsis without a defined focus”. CFR was defined as the proportion of patients who died within 30 days from culture, since IPD was considered to be a contributing factor also in patients who died from other reasons during this time interval. Dates of death were provided through patient records and by SCB. Mortality, defined as the number of deaths due to IPD/100,000 inhabitants/year, was calculated as the function of annual incidence and CFR.

Disease severity was estimated by ICU stay, need for mechanical ventilation, and complications (e. g. parapneumonic effusion, empyema, myocardial infarction). Episodes starting more than 168 h after admission to hospital for another reason were defined as nosocomial. All data for patients from the city of Gothenburg and 5 surrounding municipalities (population 643,620) were compared with data from the rest of the region (smaller cities and countryside). Long term trends concerning incidence, CFR and mortality were explored by comparing the data from Gothenburg with surroundings with data from two previous studies from the exactly the same area [5, 18], using the same study protocol, by the same principle researcher (BT), giving a total follow up time of 45 years.

In order to calculate incidence in specific risk groups, prevalences of several comorbidities were estimated as follows: Exact data on the number of HIV and dialysis patients were provided by the Department of Communicable Disease Control and Prevention, Västra Götaland Region, and the Swedish Renal Registry, respectively. Prevalence of monoclonal gammopathy of undetermined significance (MGUS), asplenia, rheumatoid arthritis, systemic lupus erythematosus (SLE) and diabetes mellitus was estimated based on data from prevalence studies of these conditions [19–23]. Two patients, who developed myeloma within two years from their IPD episode, were classified as having MGUS since this condition has been shown to precede myeloma [24]. Prevalence of malignant diseases was estimated on data from 2008 provided by The National Board of Health and Welfare (http://www.socialstyrelsen.se/statistik/statistikdatabas/cancer). The definition of Chronic Obstructive Pulmonary Disease (COPD) included emphysema and chronic obstructive bronchitis. Patients with both asthma and COPD were categorized only as COPD. Prevalence of asthma was estimated to be 8.3 % in adults (16–75 years) [25], whereas asthma prevalence in the age groups 0–15 years and above 75 years, and COPD prevalence above 16 years was estimated to be 9, 7 and 4 %, respectively, from unpublished data (Bo Lundbäck, Gothenburg University, personal communication). All asthma and COPD prevalence estimates were based on large studies conducted in the same area as the present study and during approximately the same time period. We abstained from estimating IPD incidence among patients with cardiovascular diseases, liver disease and alcohol abuse due to difficulties to define prevalence rates corresponding to our case definitions but these conditions are included among comorbidities.

Descriptive statistics (means, annual incidence rates, CFR and mortality rates) were calculated using Excel. The total number of blood cultures in each laboratory and the number of inhabitants in the region for each year were included in a linear model, in order to assess if there was an increasing number of blood cultures per inhabitant during the period. Medians were compared using Mann–Whitney *U* test. Relative risk and confidence intervals for relative risks were calculated using CIA version 2.1.2 [26]. All other statistical analyses were performed using SPSS version 19.0.0. Relative risks were calculated in univariate analyses as follows: the risk to die among patients with a certain manifestation or predisposing factor was compared to the risk to die among patients without this manifestation or predisposing factor, and the risk to get IPD among people living with a certain predisposing factor was compared to the risk among all inhabitants without that factor. In order to estimate odds ratio (OR), a multiple logistic regression model was constructed with death within 30 days as outcome and asthma, age, sex and comorbidity as explanatory variables. The comorbidity variable was constructed with respect to all predisposing factors other than asthma. The confounders that were included in the multiple logistic regression model were selected as being known as important from earlier studies. The number of events (case fatality) was 294 which allowed us to include additional variables in the multiple model (according to criterion 10:1, events per included variable). Two-tailed Fisher’s test was used to compare proportions.

## Results

### Study population

Altogether 3101 episodes of IPD were diagnosed in 1996–2008. Of these, 121 afflicting non-permanent residents were excluded. Complete clinical data were available in 2977 of 2980 episodes (99.9 %) afflicting 2885 permanent residents aged 0–101 years. Only age and gender were known for the remaining three. Mean and median ages were 60.6 and 65 years, respectively. Altogether 1483 episodes occurred in men and 1494 in women.

Pneumococci were isolated from blood alone in 2756, from CSF alone in 40, from blood and CSF in 111, from another sterile site alone in 36, and from blood and another sterile site in 34 episodes.

1. IPD incidence related to age and sex
  The mean IPD incidence was 15.1/100,000 inhabitants/year. The number of blood cultures per inhabitant per year in the region doubled during the study period; still, yearly IPD incidence (range 12.8–18.5) did not show any increasing nor decreasing trend (data not shown). The annual IPD incidence was significantly higher in all age groups compared to the group 2–17 years old: it was highest among people ≥65 years (45/100,000), followed by infants 0–23 months (23/100,000) (Fig. 1).

**Fig. 1:**
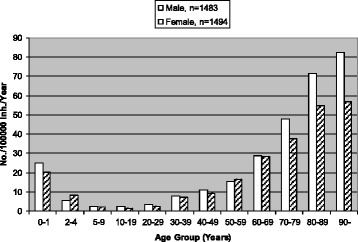
Age-specific incidence rates of invasive pneumococcal disease in men and women in different age groups
2. Comorbidity.
  The patients suffered from one or more comorbidities in 1994 (67 % of all) IPD episodes (Table 1). The most common were cardiovascular and pulmonary disease, followed by malignancies and diabetes. Altogether 983 patients did not have any comorbidity. Vaccination status was known in 720 patients. Only 67 were known to have been vaccinated against pneumococci, almost all with PPV-23.
3. Manifestations
  Pneumonia was the most common manifestation, followed by septicemia with unknown focus and meningitis (Table 2). A variety of more rare manifestations were observed (Table 3). The distribution of manifestations varied greatly with age. Among infants younger than 2 years, 31 % had meningitis compared to 5 % among elderly ≥65 years, whereas pneumonia occurred in 25 % of infants and 79 % of elderly. The annual meningitis incidence was 1.1/100,000. It was highest in children 0–1 years old, 7.0/100,000, and 2.0/100,000 among people ≥65 years. The annual incidence of non-meningitic infections was 14.1/100,000. It was highest among people ≥ 65 years, 42.8/100,000 and 15.5/100,000 among 0–1 year old children. The incidence of bacteremic pneumonia alone was 11.5/100,000/year.

**Table 2:** Manifestations in 2977 children and adults with invasive pneumococcal disease: proportions, age distribution and relative risk to die

	No. (%) of all Episodes in different age groups					Fatalities			
Manifestation^a^	0–1 y.	2–17 y.	18–50 y.	51–64 y.	65–101 y.	All ages	Died No.	CFR %	RR risk of death (95 % CI)
Septicaemia with no detectable focus	31 (32)	26 (26)	39 (7)	63 (9)	180 (12)	339 (11)	70	21	2.43 (1.91–3.10)^b^
Meningitis	30 (31)	16 (16)	42 (7)	47 (7)	73 (5)	208 (7)	28	13	1.40 (0.97–2.01)
- without pneumonia	30	16	38	33	52	169	23	14	1.41 (0.95–2.10)
- with concurrent pneumonia^a^	0	0	4	14	21	39	5	13	1.30 (0.57–2.98)
- with concurrent acute otitis media^a^	1	2	12	10	13	38	1	3	0.26 (0.04–1.83)
Pneumonia without meningitis	24 (25)	46 (46)	478 (80)	519 (78)	1199 (79)	2266 (76)	188	8.3	0.56 (0.44–0.69)^c^
- with Empyema	1	2	19	19	31	72	0	0	N.A.^d^
- with parapneumonic effusion	0	1	24	21	58	104	6	6	0.59 (0.27–1.30)
Other manifestations without pneu. or men.	12 (12)	11 (11)	35 (6)	37 (6)	69 (5)	164 (6)	8	4.9	0.48 (0.24–0.95)^c^
- Other manifestation together with pneumonia or meningitis^a^	3	4	31	35	42	115	3	3	0.26 (0.08–0.79)^c^
- all patients with other manifestations.	15	15	66	72	111	279	11	4	0.38 (0.21–0.68)^c^
All episodes^a^	97	99	594	666	1521	2977	294	9.9	

^a^In 147 episodes, more than one manifestation was detectable

^b^Statistically significant higher relative risk to die than in all episodes without this manifestation

^c^Statistically significant lower relative risk to die than in all episodes without this manifestation

^d^Not applicable

**Table 3:** Age distribution of other invasive pneumococcal disease manifestations in children and adults

	No. of Episodes in different age groups					
Other Manifestations^a^	0–1 y.	2–17 y.	18–50 y.	51–64 y.	65–101 y.	All ages
Upper respiratory tract infections (URTI)						
- acute otitis media	6	7	18	14	18	63
- sinusitis	4^b^	3	18	10	5	36
- epiglottitis	0	0	3	8	4	15
- other URTI	3	3	3	4	5	18
Skin, bone, joint and soft tissue						
- septic arthritis	1	1	4	12	38	56
- erysipelas, cellulitis, phlegmone, abscess	2	0	4	10	17	33
- osteitis	1	2	2	4	7	16
Intra abdominal						
- peritonitis	0	0	5	6	2	13
- cholecystitis, cholangitis	0	0	0	1	6	7
- salpingitis, tubo-ovarian abscess	0	0	7	0	2	9
Endocarditis	0	0	3	7	6	16
Pericarditis	0	0	0	0	7	7
Endophtalmitis, pyelonephritis, purulent thyreoiditis	0	0	0	0	5	5
No of episodes^a^	15	15	66	72	111	279

^a^In 19 episodes, more than one manifestation other than pneumonia or meningitis was detectable

^b^All infants with sinusitis suffered from ethmoiditis
4. Case Fatality Rate
  CFR was 9.9 %. It increased with age (Fig. 1), and it was higher in men (12 %) than in women (9 %), *p* < 0.01, even though the median age was higher in women than in men, 67 vs 64 years, *p* < 0.001. A multivariate logistic regression analysis yielded that male sex was an independent risk factor for death, after correcting for age, comorbidity in general and cardiovascular disease and alcohol abuse in particular (OR = 1.5, 95 % CI (1.17–1.96)). The difference in CFR between sexes was most pronounced between 50 and 70 years of age.
  Median age of those who died was 81 (range 9–100) years and of those who survived 55 (range 0–101) years (*p* < 0.001). CFR was 12.9 and 3.8 % among patients with and without comorbidities, respectively (Table 1). CFR also varied with manifestation, being highest among patients with septicemia with unknown focus, followed by meningitis and pneumonia (Table 2).
5. Disease severity and complications
  Only 25 patients were treated as outpatients. Altogether 76 (3 %) episodes were hospital acquired, 24 (32 %) of them died. Length of stay (LOS), known for 2561 admissions, varied between less than one to 525 days (mean 12 days, median 7 days). In-hospital mortality was 12 % among patients with known LOS. Altogether 477 patients were admitted to the ICU, 128 (27 %) with meningitis and 292 (61 %) with pneumonia. The average ICU LOS was 6.8 days (range 0–104). Altogether 202 patients were treated with mechanical ventilation. CFR was 18 % and in-hospital mortality 21 % among ICU patients.
  One or more specified complications occurred in 298 episodes. In 31 of them the patient died. Parapneumonic effusion was the most common (104), followed by empyema (70) and myocardial infarction (34). Multiple organ dysfunction, acute renal failure, adult respiratory distress syndrome, stroke and secondary nosocomial infections occurred in 10–20 patients each, often more than one during the same episode. Among 180 patients who survived meningitis, 70 (39 %) had sequelae. Neurologic sequelae, such as paresis and/or hearing loss dominated. Among 37 children <5 years with meningitis, one died and sequelae developed in 10, whereas 18/73 patients ≥65 years died and 25/73 had sequelae.
6. Geographical differences
  Altogether 1083 IPD episodes occurred in Gothenburg and 5 surrounding municipalities, giving an annual incidence of 12.9/100,000, compared to 1894 episodes and 16.8/100,000 in the surrounding region (*p* < 0.001). CFR was 9 % (93/1083) Gothenburg with surroundings and 11 % (201/1894) in the region (*p* = 0.08). The proportion of patients ≥80 years was lower 201/1083 in Gothenburg compared to 426/1894 in the region (*p* = 0.012). Incidence in this age group was 48 and 71/100,000/year, respectively. Age specific CFR was identical in both areas (data not shown). The distribution of underlying conditions was similar except that 12 % of the patients from Gothenburg with surroundings were known to be alcohol dependent compared to 5 % in the region (*p* < 0.001).
7. Long-term trends during 45 years: comparison with two earlier studies
  Neither incidence nor CFR did show any trend during the study period 1996–2008 (data not shown). In contrast, annual incidence of all IPD had increased from 5.3/100,000 to 10.3/100,000 from 1964–1980 to 1981–1995 (*p* < 0.01), and it had increased further to 12.9/100,000 in 1996–2008 (*p* = 0.03) (Table 4). The annual meningitis incidence remained stable, 1.1/100,000 compared to 1.3 and 1.4/100,000 earlier. Age specific incidence among infants (<2 years) did not change significantly, whereas annual incidence among persons ≥65 years had increased from 11/100,000 in 1964–1980 to 30/100,000 in 1981–1995 (*p* < 0.001), to 40/100,000 (*p* = 0.05), in the present study. The male female ratio was 1:1, compared to 2.1:1 and 1.3:1 in 1964–1980 and 1981–95, respectively. The proportion of patients with any underlying disease had remained stable during 45 years (68–72 %). In contrast, the proportion of patients with known alcohol dependency had decreased from 27 % in 1964–1980 to 12 % in 1981–1995, whereas this proportion remained the same in 1996–2008.

**Table 4:** Incidence, case fatality rate and mortality in invasive pneumococcal disease in greater Gothenburg during 45 years

	1964–1980 (17 years)					
Age (years)	Mean pop.^a^	No. IPD	Incidence^b^	Died (No.)	CFR (%)^c^	Mortality^d^
0–1	15,248	52	23	8	15	3.1
2–17	118,570	32	2	3	9	0.1
18–50	264,282	161	4	28	17	0.6
51–65^e^	98,657	129	9	27	21	1.6
66–101^e^	79,387	134	11	34	25	2.5
All ages	576,143	508	5	100	20	1.0
	1981–1995 (15 years)					
Age (years)	Mean pop.^a^	No. IPD	Incidence^b^	Died (No.)	CFR (%)^c^	Mortality^d^
0–1	15,169	67	29	5	7	2.2
2–17	103,624	35	2	3	9	0.2
18–50	280,287	189	4	18	10	0.4
51–64	84,475	167	13	27	16	2.1
65–101	99,233	441	30	83	19	5.6
All ages	582,789	899	10	136	15	1.6
	1996–2008 (13 years)					
Age (years)	Mean pop.^a^	No. IPD	Incidence^b^	Died (No.)	CFR (%)^c^	Mortality^d^
0–1	11,636	37	24	1	3	0.66
2–17	118,604	31	2	1	3	0.06
18–50	308,938	257	6	9	4	0.22
51–64	102,280	228	17	10	4	0.75
65–101	102,161	530	40	72	14	5.4
All ages	643,620	1083	13	93	9	1.1

^a^Mean population during the period. Values for 1964–1980 are calculated on data from 1968–1980

^b^Incidence: No. of IPD episodes/100,000 inhabitants/year

^c^Case fatality rate

^d^No of patients who died from IPD/100,000 inhabitants/year

^e^Data from 1964–80 concern 51–65 and 66 years and above
  CFR had decreased to 9 % from 15 % in 1981–95 (*p* < 0.001), when it had decreased from 20 % in 1964–80 (*p* = 0.03). During the last 13 years, 13 % died within 24 h of culture compared to 25 % in both previous studies. CFR among meningitis patients was 13 %, compared to 33 % in 1964–1980.
  Mortality, was significantly lower in the age groups 18–50 and 51–64 years in 1996–2008 compared to 1964–1981, whereas it had increased significantly among patients aged 65 years and above (Table 4), all p-values < 0.05. There was also a decreasing tendency in the age group 0–1 years.
8. Relative risks for patients with specific comorbidities
  The estimated annual IPD incidence varied widely between specific risk groups (Table 2). It was highest among myeloma patients, followed by patients with chronic lymphatic leukemia, hemodialysis, lung cancer, HIV, SLE, asplenia, rheumatoid arthritis, COPD, and diabetes mellitus. The CD4 count at the time of infection was known for all HIV patients; all but one had <270 cells/μL, and 7/13 had < 200 cells/μL. Asthma without COPD did not lead to an increased incidence.
  The risk to die within 30 days for patients with any comorbidity was 3.4 (2.45–4.81) times higher than for patients without. The highest relative risk to die was seen among patients with solid tumors (especially lung cancer), followed by patients with hemodialysis, cardiovascular disease and hematological malignancy, especially myeloma. CFR was significantly lower among asthma patients (RR = 0.27 (95 % CI 0.10–0.71) compared to all patients without asthma. A multiple logistic regression model considering age, sex and comorbidities, showed that the risk to die was still significantly lower among asthma patients compared to IPD patients without asthma, OR = 0.35 with 95 % CI (0.12–0.96). Only four asthma patients died; three suffered from other comorbidities and the fourth was 90 years old.
9. Multiple episodes
  Altogether 69 patients suffered from two episodes, 7 patients had three and two had four episodes. At the time of the first episode their age ranged between 1 and 89 years (mean 62 and median 68). All of them except one suffered from at least one comorbidity. Most common was hematologic malignancy which was present in 30 patients, 16 of them with myeloma. Six asplenic patients had 2–3 episodes each, all of them splenectomized because of hematologic disease. Of 78 patients with >1 episode, 10 died.

## Discussion

It is well known that the IPD incidence is highest among the elderly and small children. Although small children suffered from a substantial burden of disease in the present study, the incidence was considerably lower than among elderly, despite the fact that the study was performed prior to introduction of PCV in the childhood vaccination program. The annual incidence among children aged 0–1 years, 23/100,000, was low compared to many other countries prior to PCV vaccination, although large variations are seen between populations at a comparable socioeconomic level [13, 27]. The relatively low incidence in this age group might depend on blood culturing habits, since blood cultures are usually only performed in patients clinically evaluated as needing antibiotics intravenously. All children in this study were in-patients, compared to a study from the US, showing an annual incidence of 188/100,000 among children <2 years prior to PCV vaccination; only 29 % were in-patients [13]. Furthermore, since the blood volume obtained for culture often is suboptimal in children undiagnosed IPD might be more common among children.

A majority of all IPD episodes in this and other studies occurred in patients with previously well-described risk factors [3–5, 18, 28], and the CFR varied widely between risk groups. Death was extremely rare among patients below 45 years without any comorbidity. Because we did not have reliable data to estimate the size of the population without any risk factor, we calculated the relative risk to get IPD for each risk factor compared to all other patients, which may lead to an underestimate compared to a US study [3], that estimated the relative risk among adults with a comorbidity compared to healthy citizens.

The distribution of manifestations varied with age. For example only 25 % of 0–1 year old children with IPD had pneumonia, which is similar to studies from Switzerland and Japan [29, 30]. Notably, as many as 10 % suffered from more rare IPD manifestations, such as septic arthritis, peritonitis, endocarditis, was also more than previously described.

A CFR of 9.9 % is low compared with the earlier studies from the same area [3, 4, 28]. This is remarkable, since the proportion elderly was found to be high. CFR varied between manifestations; it was highest among patients with septicemia with unknown focus, compared to all other manifestations, probably because a high proportion of these patients had comorbidities. The CFR was also higher among meningitis patients, as expected. Complications and sequelae were also common, especially among meningitis patients, emphasizing the need for prevention through vaccination.

It is well-known that IPD epidemiology is influenced by age distribution and socioeconomic factors, and it is therefore not surprising that we found differences between the city of Gothenburg with suburbs and the rest of the region, where a greater proportion live in small towns and rural areas. The incidence in Gothenburg was lower, which was probably attributable to the fact that the proportion elderly ≥80 years was significantly lower both among IPD patients and in the whole population. However, age-specific incidence among inhabitants ≥80 years was also lower in Gothenburg compared to the rest of the region, 48/100,000/year compared to 84/100,000/year (*p* < 0.001). This might reflect that nursing home patients were less often referred to hospital and blood cultured in the large city than in the countryside. Another important finding was that alcohol abuse was more common among patients from the large city. This could have been expected to contribute to an increased total incidence in the Gothenburg area but paradoxically did not.

The increase in IPD incidence during 45 years was probably largely influenced by increased blood culturing frequency and improved culturing techniques. However, during the last 13 years, there was no further large increase in incidence despite the fact that the number of blood cultures per capita in the region doubled. Other contributing factors to the increase in IPD incidence could be increased life expectancy. In Sweden life expectancy increased between 1964 and 2008 from 74 to 80 years (www.scb.se). Survival has also increased in many serious diseases known to predispose to IPD. Finally, different clones within the same serotype may have different virulence. A new clone of serotype 1 which mainly causes IPD in healthy adults emerged in Sweden during the study period [31]. The long-term decrease in CFR is probably influenced by the increased blood culturing detecting more uncomplicated cases, but better intensive care probably also contributes to the decrease.

Meningitis incidence, however, remained rather stable during 45 years, with a slight decline during the last period. The CFR in meningitis patients declined from 33 to 13 %. Since there are no low symptomatic cases of meningitis, the incidence and CFR are less influenced by culturing practices, and more likely to be closer to the “true” incidence and CFR. CSF cultures are almost always performed, unless there are medical contraindications, and when a lumbar puncture cannot be performed, blood cultures usually give the diagnosis. The “true” incidence of pneumococcal bacteremia can only be estimated by an ideal study wherein all patients in a defined population with any symptom that possibly could be caused by a pneumococcal infection would be extensively blood cultured prior to antibiotic therapy.

Notably, the male female ratio decreased from 2.1:1 to 1:1 from the sixties until present. One contributing reason is probably a decrease in the proportion of alcoholics, which was found to be the factor with the most unequal sex distribution, 11 % of men versus 4 % of women in the present study. Smoking habits may also contribute, since the proportion smokers decreased much more among men than among women during the same period. However, this gender equalization cannot solely be explained by a decline in the proportion of alcoholics and smokers, which is underlined by the fact that it also occurred among the youngest children. In contrast, CFR remained significantly higher among men than among women, even after correcting for age and comorbidity.

IPD mortality among children 0–23 months was much lower in this study compared to the two earlier studies, and much lower than in most parts of the world. International experiences from general childhood vaccination give hope of success [12]. However, concerns that serotype replacement might erode the gains in a longer time scale [13, 32–34], underline the need for reliable baseline data before vaccination is started. Although vaccination of small children provides some protection to unvaccinated adults with increased risk through herd effects, this does not replace the need for vaccination of adult risk groups.

The highest annual IPD incidence was seen among patients with multiple myeloma: 2238/100,000, which was 154 times higher than for persons without myeloma. This was even higher than previously described [35]. PPV-23 has been shown to be poorly immunogenic in myeloma patients [36], and it remains to be shown if PCV is more effective. Since new treatment strategies have led to increased survival the need to prevent IPD in this group has increased. Recurrent infections were also common among myeloma patients, as described previously [37]. Myeloma is always preceded by MGUS [19], known to predispose for bacterial infections [38]. However, we were not able to show increased IPD incidence among MGUS patients, probably because many patients with this condition are undiagnosed. That patients with chronic lymphatic leukemia and lung cancer had a very high IPD incidence corresponds to previous studies [35]. The same is true for patients with renal disease, especially those with hemodialysis [4].

Only 13 of 3000 IPD episodes occurred in HIV positive patients. All of them except one had low levels of CD4-cells. IPD incidence is known to be elevated among HIV patients when CD4 counts are <200 cells/μL [39]. It has declined following introduction of highly active anti-retroviral therapy (HAART) [40]. Our findings were expected, since, at time of the study, HAART therapy was available for free and only 0.03 % of the inhabitants in the area were known to be HIV positive.

The estimated incidence of IPD was significantly lower among persons with asthma compared to persons without asthma. Furthermore, CFR among asthma patients with IPD was also lower than among patients without asthma, even when adjusting for age and comorbidities. This finding is easily explained by the fact that very few of the patients (54/1521) in the age group with the highest IPD incidence and highest CFR (≥65 years), were reported to have asthma. In the age group 2–49 years, no difference in incidence between persons with and without asthma was found. This finding contrasts to a study from Tennessee, US, where asthma was shown to be an independent risk factor for IPD in this age group [41]. Altogether 114 (18 %) of 635 IPD cases and 516 (8.1 %) of 6350 controls in that study had asthma. Asthma became included as an indication for pneumococcal vaccination as a result of that study [42]. The most probable explanation for why asthma was a risk factor for IPD in the US but not in the present study is differences between the study populations. The proportion of patients with coexisting factors higher in the American study, the American study primarily comprised persons of low socioeconomic status enrolled in a Medicaid program, whereas the Swedish study is population based. Furthermore, the asthma patients in the American study were not on optimal supportive treatment, illustrated by the fact that 8 % of IPD patients with asthma had received long-term oral corticosteroids and 25 % had been hospitalized for asthma within the previous year. In contrast, prevalence of respiratory symptoms was low among Swedish asthma patients during the same period, according to a study performed in the same area [25]. Furthermore, different case definitions may contribute to different conclusions, since use of asthma medication was part of the case definition in the American study but not in the present. Some of the patients classified as asthma in the US study would probably have been classified as COPD in our study, since asthma medication often is used by COPD patients. Thus, our findings do not exclude the possibility, that certain subgroups of asthma patients, such as patients with insufficient supportive treatment and contributing medical and socioeconomic factors run an increased risk to develop IPD. However, our conclusion is that asthma without COPD is not an independent risk factor for IPD in a population with good availability of medical care.

A strength of the retrospective method used is that it gave a close to 100 % coverage of all consecutive IPD patients in the area during 13 years. The success of this design relies on the quality of data in the medical records. In Sweden, medical records usually contain very detailed information on manifestations, underlying diseases and outcome. A weakness is that vaccination status only was mentioned in a minority of medical records. Furthermore, there is no centralized registration concerning the vaccination status of each individual, and sales statistics of vaccines are not official. We are still convinced that PPV-23 was largely underused in Sweden, as shown in a study from another part of the country [43]. Another weakness is that smoking and alcohol abuse might not be mentioned, so the impact of these factors may be underestimated. The impact of celiac disease, which has been shown to predispose for IPD [43], is also probably underestimated, since it is often not mentioned even when it is known.

The high incidence and mortality among elderly and in certain risk groups make vaccination a high priority, provided immunogenicity can be shown in these groups. Vaccination of infants, is probably also of benefit to risk groups, because the conjugated vaccines decrease carriage in infants and young children thereby protecting the elderly and risk groups through herd immunity. Continuous surveillance of serotype distribution among IPD cases is necessary to be able to estimate the potential benefits of vaccination of risk groups, since herd immunity may lead to an increased proportion of IPD caused by non-vaccine types [44, 45]. Hopefully, new PCVs protecting against more serotypes, or even pneumococcal vaccines providing serotype independent protection will be developed in future, in order to further reduce the high burden of disease caused by IPD.

## Conclusion

This study, together with two earlier studies, performed in the same area using the same protocol, gives a unique insight in how pneumococcal epidemiology has developed without impact of vaccination during 45 years. These findings may also be valid for other countries at a comparable socioeconomic level. The study creates the baseline needed for a future evaluation of PCV in the general childhood vaccination program, because detailed information on manifestations, death, severity, complications and risk groups is needed to be able to fully understand the effects of vaccination. This study provides important baseline data which have to inform immunization policy.

## Abbreviations

CFR, case-fatality rate; COPD, chronic obstructive pulmonary disease; CSF, cerebrospinal fluid; HAART, highly active anti-retroviral therapy; IPD, invasive pneumococcal disease; MGUS, monoclonal gammopathy of undetermined significance; OR, odds ratio; PCV, pneumococcal conjugate vaccines; PPV, polysaccharide vaccine; SCB, Statistics Sweden; SLE, systemic lupus erythematosus
